# Neural activity during processing Chinese irony text: An event-related potential study

**DOI:** 10.3389/fnins.2022.1019318

**Published:** 2022-10-05

**Authors:** Hanwen Shi, Yutong Li

**Affiliations:** School of Psychology, Liaoning Normal University, Dalian, China

**Keywords:** irony, literal, predictability, ERP, N400

## Abstract

**Objective:**

Irony as an indirect language with unpredictability consumes more cognitive resources, and is more difficult to understand than literal language. This study aims to explore the processing differences between irony and literal sentences using event-related potential (ERP) technology.

**Materials and methods:**

Three types of sentences were involved: sentences with predictable literal meaning, sentences with unpredictable literal meaning, and sentences with ironic meaning. The neural responses of the subjects were recorded when they read sentences.

**Results:**

Compared to predictable literal meaning sentences, unpredictable literal meaning sentences and ironic meaning sentences elicited larger amplitude of N400 components. The difference was not significant between the latter two. In addition, there was no significant difference in P600 evoked by the three sentences.

**Conclusion:**

In the initial stage of irony processing, the low predictability may result in the difficulty in semantic comprehension, in which the processing patterns of unpredictable and ironic sentences are rather close. In the later stage of processing, ironic integration is not harder compared to literal sentence integration.

## Introduction

Irony is a kind of indirect language characterized as contrary to its literal meaning, reflecting the speaker’s potential attitude of humor, criticism, or mocker (Hancock et al., 2000). The understanding of irony requires combination with the specific context. The outcomes of behavioral research are inconsistent as to whether the processing of irony and literal meaning is differential, as some studies found no temporal difference (Gibbs, 1986), while some found longer time taken by processing irony (Dews and Winner, 1999). Many studies have explored neural responses to processing irony using event-related potentials (ERPs), generally focused on N400 and P600 components associated with language processing (Regel et al., 2010; Filik et al., 2014; Caffarra et al., 2019).

The N400 was a negative component found in the frontal region from 300 to 500 ms after stimulus onset (Kutas and Federmeier, 2011), and the P600 was a positive component in the central frontal region from 500 to 800 ms after stimulation onset (Osterhout and Holcomb, 1992). The semantic retrieval hypothesis holds that the N400 component reflects the difficulty of semantic information retrieval (Brouwer et al., 2012). The irony is more difficult to understand in comparison to the literal meaning, the N400 amplitude was correspondingly larger (Li et al., 2020). Cornejol et al. (2007) explored the effect of participants’ processing strategies on processing irony, reporting that the N400 amplitude was larger in the ironic condition in comparison to the literal condition when the holistic processing strategy was applied. Filik et al. (2014) had subjects listen to ironic sentences with varying familiarity, with their EEG (electroencephalograph) changes recorded. The results showed that the amplitude of N400 elicited by the ironic condition was larger in comparison to the literal condition when processing unfamiliar sentences. However, when exposed to sentences in a natural or ironic intonation (Balconi and Amenta, 2008), there was no N400 difference between the ironic intonation and the natural intonation. Regel et al. (2011) asked subjects to read and listen to the literal sentence and the ironic sentence, finding no difference in N400 amplitude between the ironic condition and the literal condition, which is also obtained by Spotorno et al. (2013). In sum, the results of processing irony and processing literal sentences are inconsistent in N400 amplitude changes according to the above EEG experiments. The difficulty of semantic processing of irony does not keep unchanged, which may be affected by sentence familiarity, processing strategy, or other factors. The N400 effect cannot serve as a key of irony processing (Spotorno et al., 2013). P600 reflects the process of structural repair and reanalysis in sentence comprehension (Friederici, 2002), which is sensitive to a variety of syntactic changes (Hagoort, 2003), and appears for grammatically complicated or ambiguous sentences (Friederici, 2002), as well as semantically and syntactically normal sentences with more complex pragmatics and concepts (such as irony, rhetoric, and jokes) (Coulson and Kutas, 2001; Spotorno et al., 2013). Research has indicated that irony elicits a larger P600 amplitude compared to literal meaning (Caffarra et al., 2019). In the late stage of processing, more cognitive resources are required to process and integrate irony. The P600 changes in irony processing are modulated by speaker styles. Regel et al. (2010) have manipulated the speaker styles, one for irony speakers (70% of their comments are ironic) and the other for non-irony speakers (70% of their comments are literal), finding a more significant P600 amplitude evoked by the ironic comments of non-irony speakers in comparison to the literal comments, without obvious difference revealed in P600 between ironic and literal comments of ironic speakers. A study has demonstrated that the subsequent ironic and literal evaluations of ironic characters could be equally processed quickly (Turcan et al., 2020). These outcomes may be resulted by the pragmatic expectations, in which the tendency of ironic speakers to give out ironic comments enhances the predictability of irony and affects the semantic processing. When irony can be predicted, fewer integration resources are occupied, and processing irony does not necessarily induce a larger amplitude P600 (Akimoto et al., 2017).

In conclusion, the temporal course of irony processing still remains uncovered, which involves both N400 and P600 components. Some studies have revealed the difficulty in semantic extraction and the increased requirement of cognitive resources in the late integration stage of irony processing. However, other studies have indicated similar EEG patterns evoked by irony processing and literal sentence processing. The contribution for this bias may be the factors of experimental tasks, variables operated by the experimenter, and predictability. Both P600 and N400 effects can be interpreted as indicators of prediction errors (Fabry, 2021). Among them, the predictability is a critical factor generally ignored by researchers. With the analysis of indirect evidence, we identified a close relation of predictability to irony processing, in which the predictability potentially plays a certain role in the difficulty of irony processing. Up to now, there has not emerged ERP study to directly explore the role of predictability in irony processing.

Ironic sentences are less predictive than literal ones, and whether the difficulty in understanding irony is related to low predictability remains uncovered. In fact, language comprehension is a procedure of processing lexical information according to one’s own knowledge, which requires continuous prediction of the incoming language information accompanied with continuous adjustment with the evaluation of the prediction results, so as to reduce the possibility of further prediction errors. This is also the view holden by predictive coding theory (Friston, 2005, 2010), that is, prediction is performed from top to bottom with the wrong prediction adjusted according to bottom-up information. Fabry (2021) explains irony processing depending on predictive coding theory, that understanding ironic sentences produced more predictive errors in comparison to literal sentences. Errors in predicting irony lead to the reprocess of it until the error is corrected and the irony is understood.

Studies have indicated a prominent role of the left ventral occipitotemporal region in visual word recognition (Dehaene and Cohen, 2011; Vogel et al., 2013), which also significantly contributes in perceptual reasoning, receiving top-down predictions from brain regions associated with phonological, semantic, and lexical processes (Carreiras et al., 2014). EEG experiments support a link of language processing with predictability. The N400 component seems to be associated with the small probability of words emerging in the background (Frank et al., 2015; Kuperberg and Jaeger, 2016). Fabry (2021) holds that background information as a prior knowledge is encoded in the process of generating prediction information from top to bottom. Based on this, the individual will constantly adjust the processing process, achieving to avert the prediction error, and understand the irony. However, there is no direct evidence to support the relationship between the two processing courses. As a result, in this study, sentence types were manipulated to examine differences in the processing of predictable literal, unpredictable literal, and ironic sentences. Under the condition of predictable literal meaning, the literal meaning of remark is the meaning that the speaker wants to express, and remark sentences are predictable. Under the condition of unpredictable literal meaning, however, remark sentences are not predictable. Under the irony condition, the literal meaning of remark sentences is opposed to the meaning that the speaker wants to express, and remark sentences are not predictable.

The encoding irony in the early stage is different from that in late stage (Fabry, 2021). This study hypothesized that irony and unpredictable literal conditions lead to more significant neural responses in comparison to predictable literal conditions during the early stage of processing. We anticipated a greater N400 amplitude elicited the irony and unpredictable literal conditions compared to the predictable literal conditions during the early semantic extraction phase. According to Fabry’s (2021) suggestion that irony processing difficulties are linked to expectation violation, we hypothesized no difference in N400 amplitude lying between irony and unpredictable literal conditions. With the further processing, readers can find that irony expresses the non-literal meaning in accordance with the context. Understanding irony requires a longer chain of reasoning compared to predictable literal sentences, and the difficulty of integrating unpredictable literal sentences does not disappear after context integration. Therefore, this study hypothesized that, in the later stages of processing, both the irony and the unpredictable literal condition could evoke a more significant neural response in comparison to the predictable literal condition, while unpredictable led to a more significantly stronger one than irony condition. Specifically, due to the increased integration requirements of ironic and unpredictable sentences, unpredictable literal condition and irony condition correspondingly produce larger P600 amplitude compared to predictable literal conditions in the late integration phase (P600), and unpredictable literal conditions produce larger P600 amplitude than ironic conditions.

## Materials and methods

### Participants

Thirty-three students (19 females; average 23.1 years old) participated in the experiment. All participants were native Chinese speakers, equipped with normal or corrected-to-normal vision without reading disorders. The participants provided written informed consent and were financially compensated for their participation. The protocol of the experiment was reviewed and approved by the Ethics Committee at the College of Psychology, Liaoning Normal University.

### Materials and design

The experimental material was composed of two parts: context and target sentence. The material covered a total of 120 contexts and 40 target sentences. Each context was composed of two sentences, describing a scene involving two characters. The target sentence was a remark. Three different contexts were constructed for each target sentence, producing three conditions combined with different context sentences: (a) a predictable literal remark; (b) an ironic remark; and (c) an unpredictable literal remark. All target sentences were divided into three segments, with the last segment as the critical segment and the critical segment as an adjective. Three experimental conditions were applied in this study, the predictable literal condition (PL), the unpredictable literal condition (UL), and the ironic condition (IR). Examples of experimental materials are listed in Table 1.

**TABLE 1:** Example material.

Condition	Context	Remark sentences
Literal	张进安装软件出现问题, 于是去找范博帮忙。 范博帮他找各种解决办法。张进说道: Zhang Jin had problems installing the software, so he went to Fan Bo for help. Fan Bo looked for various solutions to help him. Zhang Jin said:	“你真是**热心**” “you are so **warm-hearted**”
Irony	张进安装软件出现问题, 于是去找范博帮忙。 范博草草看了一眼就说自己也无能为力。张进说道 Zhang Jin had problems installing the software, so he went to Fan Bo for help. Fan Bo glanced at the software hastily and indicated that he could not help. Zhang Jin said:	“你真是**热心**” “you are so **warm-hearted**”
Unexpected	张进安装软件出现问题, 于是去找范博帮忙。 范博正好从食堂回来。张进说道: Zhang Jin had problems installing the software, so he went to Fan Bo for help. Fan Bo just came back from the cafeteria. Zhang Jin said:	“你真是**热心**” “you are so **warm-hearted**”

### Irony norming

To investigate the processing diversity between Chinese irony sentences and different predictive literal meaning sentences, the irony condition, predictable literal condition, and unpredictable literal condition were set, respectively. Following the method proposed by Turcan and Filik (2016), 17 volunteers were invited to rate the irony of the experimental materials on a scale of 1−8. The results of one-way analysis of variance (ANOVA) revealed significant differences among the three groups of scores, *F*(2, 48) = 295.50, *p* < 0.001, η_*p*_^2^ = 0.93. The results of multiple *post-hoc* comparisons indicated the significantly higher score reported in the ironic condition in comparison to the unpredictable literal condition [*t*(50) = 21.17, *p* < 0.001, *Cohen’s d* = 7.10]. The score of the predictable literal condition was reported significantly decreased in comparison to the irony condition [*t*(50) = −20.94, *p* < 0.001, *Cohen’s d* = −6.95], without significant difference between unpredictable and predictable literal conditions [*t*(50) = 0.23, *p* = 1.00, *Cohen’s d* = 0.08] [*M*_*IR*_ = 6.80, *SD* = 0.74; *M*_*UL*_ = 1.95, *SD* = 0.62; *M*_*PL*_ = 2.00, *SD* = 0.64]. *P*-values have been corrected with Bonferroni.

### Cloze norming

In order to verify the predictability of the three experimental conditions, an online cloze test was performed on the adjectives of the target sentences, recruiting 17 volunteers to perform cloze tests online, who were presented with complete experimental materials, except for a critical word in the target sentence. They were asked to complete an adjective in the sentences. Irony condition and unpredictable literal condition led to lower accuracy than predictable literal condition [*M*_*IR*_ = 0.03, *SD* = 0.03; *M*_*UL*_ = 0.03, *SD* = 0.03; *M*_*PL*_ = 0.79, *SD* = 0.13], indicating the low predictability of these two conditions. The one-way ANOVA revealed the significant differences among the three groups of scores, *F*(2, 48) = 490.89, *p* < 0.001, η_*p*_^2^ = 0.95. The results of multiple *post-hoc* comparisons demonstrated that the scores of predictable literal conditions were significantly higher in comparison to the unpredictable literal conditions [*t*(50) = 27.11, *p* < 0.001, *Cohen’s d* = 7.74] and the irony condition [*t*(50) = 27.16, *p* < 0.001, *Cohen’s d* = 7.83], without difference in the scores of unpredictable literal conditions and irony [*t*(50) = −0.05, *p* = 1.00, *Cohen’s d* = −0.05]. *P*-values have been corrected with Bonferroni.

### Procedure

**FIGURE 1:**
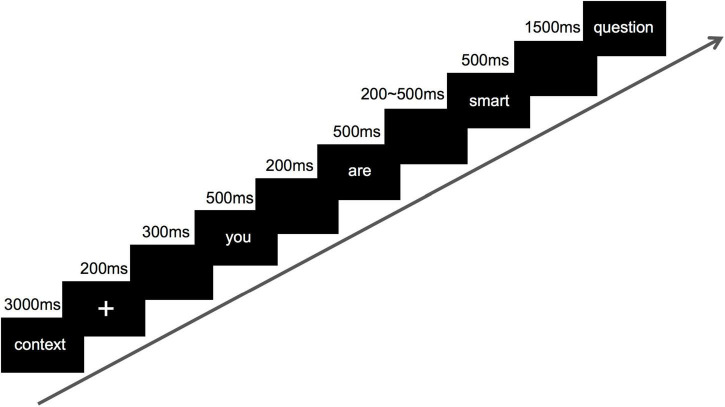
Schematic representation of the experimental design.

Participants were seated comfortably in a dimly lit, electrically-shielded chamber, and viewed the display with a distance of 57 cm. Each trial was initially initiated with a context of 3,000 ms, followed by a fixation cross of 200 ms, and an empty screen of 300 ms. Afterward, the target sentence was presented word by word, each for 500 ms, the blank screen interval between the words lasted 200 ms, and the last one was the variable blank screen interval (200–500 ms) before the last word, which was rather critical to determine the valance of comment, so the mark was placed on this word. After the target sentence ended, there followed a 1,500 ms blank screen, and then a question of whether to agree with the evaluation that just appeared. Not until the subject pressed the key (F represent agree, J represent disagree) did the screen disappear (Experimental procedure was detailed in Figure 1). There was no right or wrong in the task of the experiment, so the result of task was not analyzed. This study covered three experimental conditions, with each experimental condition composed of 40 trials. The task consisted of two blocks of 60 trials, yielding a total of 120 trials per participant.

### Electroencephalograph recording

Electroencephalograph was recorded through 64 Ag/AgCl scalp electrodes depending on the 10/20 system of electrode placement. During recording, the Auricular Frontal zero (AFz) was defined as ground and Frontal Central zero (FCz) as reference. An electrode was placed under the right eye to record the vertical ocular electro-oculogram (VEOG), with the filter bandwidth of 0.01–100 Hz. The EEG signal from each channel was digitized at a 500-Hz sampling rate. The impedance of all electrodes was kept below 10 kΩ.

### Data analysis

Electroencephalograph preprocessing was carried out on EEGLAB (Delorme and Makeig, 2004), an open-source toolbox based on MATLAB. Data was re-referenced off-line to the left-mastoid, with bad channels interpolated. Independent component analysis (ICA) was adopted to remove eye blinks, eye movements, body movements, and channel noise from the data. Data were filtered by 0.1 Hz high-pass and 30 Hz low-pass filters, respectively. Subsequently, stimulus-locked epochs of −200 to 1,000 ms were determined, and the baseline was corrected using the 200 ms window before stimulus presentation. Finally, epochs with an amplitude change exceeding 100 μV on any channel were excluded from further analysis. Nine electrode points were selected according to previous literature to define different scalp regions (Frontal: F3, F4, and Fz; Central: C3, C4, and Cz; and Parietal: P3, P4, and Pz) (Spotorno et al., 2013), on which we measured the N400 and P600 components. The time windows of the N400 and P600 components are set to 300–500 ms and 500–800 ms, respectively. To compare the differences between irony processing and unpredictable literal sentence processing, the EEG data were performed with a two-way repeated measures ANOVA, with condition (predictable literal, irony, and unpredictable literal) and brain region (frontal, central, and parietal) defined as within-subject factors. All results were subjected to Bonferroni correction for multiple tests.

In order to confirm the validities of statistical inference results, the Bayesian factor analysis was additionally carried out based on the classical statistical method, Null hypothesis significance testing (NHST). Bayes factor can be interpreted as the degree of support for null hypothesis H0 or alternative hypothesis H1, which serves as an essential method applied in model comparison and hypothesis testing in Bayesian statistics (Peng et al., 2018), where the statistical significance was excluded, but for description on the extent to which the data supports the hypothesis. The EEG data of N400 and P600 were analyzed by Bayesian repeated measures ANOVA on JASP software (JASP Team, 2022).^1^ The Bayes factor (BF_10_) can be defined as follows: 1 represents no difference, 1–3 represents weak evidence supporting H1, 3–10 represents moderate evidence supporting H1, 10–30 for strong evidence supporting H1, 30–100 for very strong evidence supporting H1, and over 100 for very strong evidence supporting H1 (Wagenmakers et al., 2018).

## Results

**FIGURE 2:**
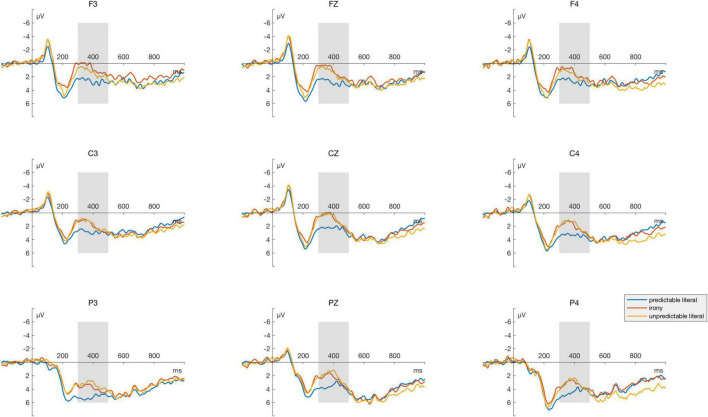
Grand average event-related potentials (ERPs) analyzed for the critical words of all conditions. Grand averaged ERPs for target stimuli at Frontal, Central, and Parietal sites. The area marked by the gray bar is the 300–500 ms interval on the time axis, which is the time window of the N400 component.

**FIGURE 3:**
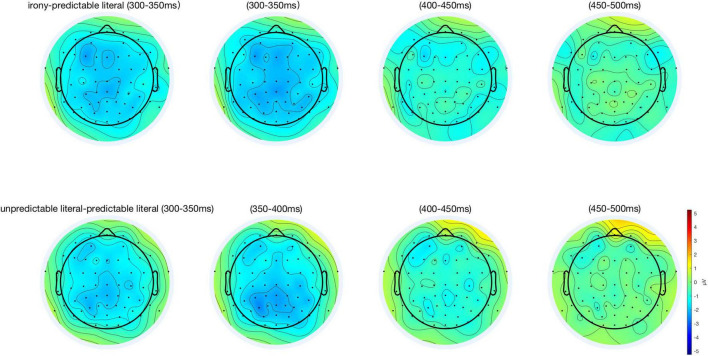
The topographic maps of the scalp distribution of the N400 effect. The **top row** contains three topographic maps evoked by the N400 amplitude of the irony condition minus the predictable literal condition, with 50 ms intervals. The **bottom row** contains three topographic maps generated by the unpredictable literal conditions minus the predictable literal conditions.

Figures 2, 3 illustrate the grand-average ERP difference waveforms and topographic maps of N400 effect between different conditions, which were recorded from nine electrodes (Frontal: F3, F4, and Fz; Central: C3, C4, and Cz; and Parietal: P3, P4, and Pz).

In the time window between 300–500 ms, the result of main analysis showed a main effect of condition [*F*(2, 64) = 4.18, *p* = 0.02, η_p_^2^ = 0.12]. Through pairwise comparison, we found a greater N400 amplitude induced irony conditions in comparison to predictable literal conditions (*t* = 2.49, *p* = 0.04, *Cohen’s d* = 0.43), and similarly, unpredictable literal conditions yield greater N400 volatility than predictable literal conditions (*t* = 2.52, *p* = 0.04, *Cohen’s d* = 0.44), without significant difference between irony conditions and unpredictable literal conditions (*t* = 0.04, *p* = 1.00, *Cohen’s d* = 0.01). There was also a significant difference between brain regions [*F*(2, 64) = 5.01, *p* = 0.01, η*_*p*_*^2^ = 0.14], without significant interaction indicated [*F*(4, 128) = 1.23, *p* = 0.30, η_p_^2^ = 0.04]. The outcomes of Bayesian factor analysis further validated the results of ANOVA, with the two primary effects models (condition + region) opposing the null model most robustly (BF_10_ = 7,299.13), which indicated that the probability of the current data under the alternative hypothesis (assuming an effect) was 7,299.13 times higher than that under the null hypothesis (assuming no effect), providing powerful evidence in favor of the alternative hypothesis.

In the time window between 500–800 ms, the ANOVA reported a significant main effect of brain region [*F*(2, 64) = 8.64, *p* < 0.001, η*_*p*_*^2^ = 0.21]. No significant effect was revealed in the condition [*F*(2, 64) = 0.21, *p* = 0.81, η*_*p*_*^2^ < 0.01] and the interaction of condition and brain area [*F*(4, 128) = 6.82, *p* = 0.61, η*_*p*_*^2^ = 0.02]. To verify the null effects of the condition variables, a Bayesian factor analysis was performed, finding that the current data were more likely to support the null hypothesis (BF_01_ = 16.45). Referring to the Bayesian factor criterion of Wagenmakers et al. (2018), the BF_01_ value of 16.45 indicated that the current data is 16.45 times more likely to occur under the null hypothesis (which assumes no effect) than under the alternative hypothesis (which assumes an effect), serving as the strong evidence in favor of the null hypothesis.

## Discussion

Event-related potential studies on irony have proved that the process of irony is more complex than literal sentences. This study explored the processing differences between irony and literal sentences with ERP technology, with three kinds of Chinese sentences defined as the experimental materials: predictable literal sentences, unpredictable literal sentences and irony. By manipulating the meaning of context sentences, the remark sentences could express different meanings. The subjects were first exposed to context sentences and then to remark sentences word by word. The results showed higher N400 amplitude when individuals processed irony and unpredictable literal sentences in comparison to predictable literal sentences, while no difference in the amplitude of P600 components elicited by the three conditions.

Both unpredictable literal sentences and irony induced the increased N400 amplitudes compared to predictable literal sentences, and the similar N400 elicited in the unpredictable literal sentences and irony conditions. This study proposes a new explanation for irony processing difficulties. Irony processing is difficult in the early stage, which is consistent with unpredictable literal sentences to some extent. It can be speculated that the difficulty in the early stage of irony processing can result from the low predictability of irony itself. It has been demonstrated that processing unfamiliar irony will induce higher N400 amplitude (Filik et al., 2014), also suggesting the relation of irony processing difficulty with unpredictability. Some studies have found that irony processing has no difficulty in retrieving semantic information, revealing no N400 amplitude difference between irony and literal sentences, when there provides sufficient background information (Balconi and Amenta, 2008; Spotorno et al., 2013), which will decrease irony processing difficulty. This suggests the predictability or familiarity as the influencing factor of irony processing. The results of this study are consistent with the predictive coding theory (Friston, 2005, 2010), that is, in the process of speech information processing, the individual compares the top-down prediction with the bottom-up input, and once inconsistent with the irony, telling the prediction is wrong, it is required to adjust until the irony is understood. The elevation in the amplitude of N400 represents the cognitive resource consumption of adjusting for prediction errors (Fabry, 2021). Adjustments are demanded when predictions appear bias, which will lead to increased neural activity. Speech processing is a serial process, where literal meaning is initially activated in the early stages of sentence processing (Palinkas, 2013). The literal meaning of irony is inconsistent with the context, thus requires further processing until the irony is understood, resulting in a larger N400 amplitude in comparison to literal sentences. The unpredictable literal meaning sentence induced a larger N400 amplitude than the predictable literal meaning sentence. Previous studies have demonstrated that the amplitude of N400 is increased when individuals process sentences that violate semantic prediction (Aurnhammer et al., 2021). N400 components are affected by semantic predictability, as unpredictable conditions where individuals cannot accurately predict the remark sentence result in a larger N400 amplitude. The most critical result was that there was no difference in amplitude between the ironic condition and the unpredictable literal condition on the N400 component, providing direct evidence supporting the prediction theory of irony understanding, proposing that language understanding is also a process of constant updating and adjusting predictions based on prior knowledge for reducing prediction errors (Fabry, 2021). From word recognition to understanding of sentence and discourse, top-down prediction and bottom-up prediction error feedback interact to update the prediction, where N400 can be considered as an indicator of predictive error in irony comprehension. That the irony condition and the unpredictable literal condition show similar N400 amplitude mode indicates that the difficulty of irony processing is resulted from the unpredictability of semantic information.

The P600 component reflects the mental representation of word construction, reorganization, and renewal, serving as an indicator of late integration. A study has found that complex sentences induce larger P600 amplitude (Coulson and Kutas, 2001), but failed to demonstrate a more significant amplitude change in P600 elicited by irony or unpredictable literal sentences in comparison to predictable literal sentences. Researcher explored the influence of speaking style on irony understanding, finding no difference in the P600 amplitude between the irony condition and the literal condition when the sarcastic speaker spoke irony (Regel et al., 2010). Researcher also failed to reveal the difference in P600 amplitude between the ironic condition and the literal condition, who argue that irony does not necessarily elicit a stronger P600 response when integration requirements are relatively low (Akimoto et al., 2017). Their study has provided rich background information covering faces to scene images. Therefore, the P600 effect may not be a typical effect of irony processing. As the P600 component is related to task difficulty and integration requirements, the context materials in this study are relatively simple and easy to understand, with the scenes in the context generally the scenes of daily life, resulting in no P600 effect observed, which may be due to the simplicity of the task. Whether it is the predictable literal condition, the ironic condition, or the unpredictable literal condition, individuals can easily complete the integration of information without too much cognitive effort.

This study investigated the EEG activity of Chinese irony processing from a predictive perspective. The results showed that irony processing difficulty mainly appeared in the early stage. Speech processing is predicated on a top-down approach, is prone to error in terms of irony, which requires to be adjusted and updated. However, irony and unpredictable literal sentences have no more significant difficulty than predictable literal sentences in the late stage of linguistic information integration. Based on the above findings, this study answers the issues of the difficulty in irony processing in an explorative manner. We believe that the difficulty in irony processing is associated with relative lower prediction of irony to literal sentences, and the impact of prediction should be considered in future research. After controlling for prediction levels of irony and literal condition, a comprehensive understanding of the processing of irony may be obtained. Although this study proposed a relationship between difficulty and predictability in irony processing, there are some limitations of this study. First, we explored whether irony and literal comprehension differed in low-prediction scenarios, demonstrating the association which was supported by data. However, the condition setting of this study is not perfectly complete, and the condition of predictable irony remains to be examined, which can make the research into a two-factor within-subject experimental design with a complete structure, also contributing to clearly and comprehensively exploring the relationship between literality and predictability. Secondly, this study did not find ERP differences between unpredictable literal and irony conditions, whether there exists a difference between the two in the time-frequency domain still remains unknown. The ERP was the extracted time-locked neural activity from EEG signals, which, however, failed to be probed for non-phase-locked activity. For example, researchers found that world knowledge violation and semantic violation produced similar activation of N400 components, while reporting a difference in time-frequency activity between these two conditions. World knowledge violation showed gamma activation, which was not found in semantic knowledge violation (Hagoort et al., 2004). Therefore, whether there exists a difference between the unpredictable literal condition and the ironic condition still requires to be explored in future research.

## Data availability statement

The raw data supporting the conclusions of this article will be made available by the authors, without undue reservation.

## Ethics statement

The studies involving human participants were reviewed and approved by the Ethics Committee of Liaoning Normal University. The patients/participants provided their written informed consent to participate in this study.

## Author contributions

HS designed the experiment and wrote the first draft of the manuscript. YL revised the manuscript. HS and YL analyzed the data. Both authors contributed to the article and approved the submitted version.

## Footnotes

1
https://jasp-stats.org/
